# The Transformative Role of Artificial Intelligence in Plastic and Reconstructive Surgery: Challenges and Opportunities

**DOI:** 10.3390/jcm14082698

**Published:** 2025-04-15

**Authors:** Masab Mansoor, Andrew F. Ibrahim

**Affiliations:** 1 Edward Via College of Osteopathic Medicine—Louisiana Campus, Monroe, LA 71203, USA; 2 School of Medicine, Texas Tech University Health Sciences Center, Lubbock, TX 79430, USA; andrew.ibrahim@ttuhsc.edu

**Keywords:** artificial intelligence, machine learning, deep learning, plastic surgery, surgical planning, robotic surgery, patient monitoring, surgical outcomes

## Abstract

**Background/Objectives**: This study comprehensively examines how artificial intelligence (AI) technologies are transforming clinical practice in plastic and reconstructive surgery across the entire patient care continuum, with the specific objective of identifying evidence-based applications, implementation challenges, and emerging opportunities that will shape the future of the specialty. **Methods**: A comprehensive narrative review was conducted analyzing the integration of AI technologies in plastic surgery, including preoperative planning, intraoperative applications, postoperative monitoring, and quality improvement. Challenges related to implementation, ethics, and regulatory frameworks were also examined, along with emerging technological trends that will shape future practice. **Results**: AI applications in plastic surgery demonstrate significant potential across multiple domains. In preoperative planning, AI enhances risk assessment, outcome prediction, and surgical simulation. Intraoperatively, AI-assisted robotics enables increased precision and technical capabilities beyond human limitations, particularly in microsurgery. Postoperatively, AI improves complication detection, pain management, and outcomes assessment. Despite these benefits, implementation faces challenges including data privacy concerns, algorithmic bias, liability questions, and the need for appropriate regulatory frameworks. Future directions include multimodal AI systems, federated learning approaches, and integration with extended reality and regenerative medicine technologies. **Conclusions**: The integration of AI into plastic surgery represents a significant opportunity to enhance surgical precision, improve outcome prediction, and expand the boundaries of what is surgically possible. However, successful implementation requires addressing ethical considerations and maintaining the human elements of surgical care. Plastic surgeons must actively engage with AI development to ensure these technologies address genuine clinical needs while aligning with the specialty’s core values of restoring form and function, alleviating suffering, and enhancing quality of life.

## 1. Introduction: The Role of Artificial Intelligence in Plastic Surgery

Plastic and reconstructive surgery has historically been at the forefront of surgical innovation, embracing new technologies to improve patient outcomes, enhance surgical precision, and expand the boundaries of what is surgically possible. In recent years, artificial intelligence (AI) has emerged as a transformative force across healthcare, with the potential to fundamentally alter how plastic surgeons plan, perform, and evaluate surgical interventions [1]. The integration of AI technologies into plastic surgery represents both a significant opportunity and a complex challenge that spans clinical practice, research methodology, and business operations.

Plastic surgery is uniquely positioned to benefit from AI applications due to several domain-specific characteristics. First, the specialty’s highly visual nature aligns with the strengths of computer vision and image recognition algorithms, which can analyze preoperative images, predict postoperative outcomes, and assess surgical results with increasing accuracy [2,3]. Second, the field’s emphasis on precision and aesthetic outcomes creates opportunities for AI to assist in quantitative analysis of subjective results [4]. Third, the diverse range of procedures—from reconstructive microsurgery to aesthetic interventions—provides rich datasets across varied anatomical regions and surgical techniques, enabling robust algorithm development and validation [5].

AI encompasses a broad spectrum of computational approaches, including machine learning (ML), deep learning (DL), natural language processing (NLP), and computer vision, each with distinct applications in plastic surgery [6]. Machine learning algorithms can identify patterns in large datasets to predict surgical outcomes or complications, while deep learning neural networks excel at image-based tasks such as surgical planning and outcome simulation [7]. Computer vision applications assist in preoperative planning and intraoperative guidance, while NLP can extract valuable insights from clinical notes and the research literature [8,9].

Current applications of AI in plastic surgery span the entire patient journey. Preoperatively, AI systems help with patient selection, risk stratification, and surgical planning through 3D modeling and outcome prediction [10,11]. Intraoperatively, AI-enhanced robotics and navigation systems improve precision and may reduce operative time [12,13]. Postoperatively, AI algorithms monitor recovery, predict complications, and assess functional and aesthetic outcomes [14]. Beyond direct clinical applications, AI is transforming research methodology through automated literature analysis and dataset curation while also reshaping business operations through optimized patient scheduling, resource allocation, and marketing analytics [15].

Despite these promising developments, the implementation of AI in plastic surgery faces significant challenges [16]. Technical barriers include the need for large, diverse, and high-quality datasets for algorithm training, as well as issues of interoperability between various healthcare systems. Ethical considerations encompass patient privacy, informed consent for AI use, algorithmic bias, and questions of liability when AI systems contribute to clinical decisions. Regulatory frameworks for AI in surgery remain in development, creating uncertainty around validation requirements and standards for clinical implementation.

As AI technologies continue to evolve, plastic surgeons must actively engage with these developments to shape how AI is integrated into clinical practice. This requires not only technical understanding but also critical evaluation of AI’s limitations and ethical implications. The future plastic surgeon will likely need competencies in data science and AI evaluation to effectively leverage these tools while maintaining the human judgment and artistic sensibility that define the specialty [17].

This review examines the current landscape of AI applications in plastic surgery, explores emerging technologies and methodologies, and discusses the challenges and opportunities that AI presents for the specialty. By providing a comprehensive overview of this rapidly evolving field, this review aims to equip plastic surgeons with the knowledge needed to navigate the AI revolution and harness its potential to improve patient care, advance research, and optimize practice management.

## 2. AI in Preoperative Planning

Preoperative planning represents one of the most promising areas for AI integration in plastic surgery [18]. The ability to accurately predict surgical outcomes, assess patient-specific risks, and optimize surgical approaches has significant implications for both patient satisfaction and clinical outcomes [19]. This section explores the various applications of AI in the preoperative phase of plastic and reconstructive procedures.

### 2.1. Patient Selection and Risk Assessment

AI algorithms have demonstrated remarkable capabilities in analyzing patient data to identify ideal candidates for specific procedures and to predict individual risk profiles [20]. These systems integrate diverse inputs including medical history, laboratory values, imaging studies, and even social determinants of health to generate personalized risk assessments [21].

Machine learning models have been developed to predict complications following breast reconstruction [22], with some studies showing superior accuracy compared to traditional clinical judgment [23,24]. These models identify patients at higher risk for specific complications such as flap necrosis, infection, or implant failure, allowing surgeons to modify their approach or implement targeted preventive measures. Similarly, in aesthetic surgery, AI algorithms can analyze patient characteristics to predict satisfaction levels or identify patients with body dysmorphic disorder who might benefit from psychological intervention before considering surgery [25].

Risk stratification tools powered by AI are increasingly being integrated into clinical decision support systems, providing real-time guidance during patient consultations [26]. These tools not only help surgeons make more informed decisions but also improve the informed consent process by generating patient-specific risk profiles rather than relying on population-level statistics [27].

### 2.2. Outcome Prediction and Simulation

Perhaps the most visible application of AI in preoperative planning is in outcome prediction and simulation [28]. Computer vision and deep learning algorithms can analyze preoperative images to generate realistic visualizations of expected surgical outcomes, helping to align patient expectations with achievable results [29].

In facial aesthetic surgery, AI systems can simulate the effects of procedures such as rhinoplasty, face lifting, or blepharoplasty based on the patient’s unique facial anatomy and the surgeon’s planned intervention [30]. These simulations go beyond simple morphing techniques, incorporating biomechanical properties of skin and underlying tissues to create more realistic predictions [31]. Some advanced systems can even account for aging processes to demonstrate long-term outcomes [32].

For breast surgery, AI algorithms can recommend optimal implant sizes and shapes based on the patient’s anatomy, desired outcome, and tissue characteristics [33]. Three-dimensional simulations allow patients to visualize potential results with different implant options, significantly improving the shared decision-making process. In reconstructive cases, these technologies help surgeons plan flap design and placement to optimize aesthetic outcomes while ensuring adequate tissue perfusion [22,34].

### 2.3. Advanced Imaging Analysis and 3D Modeling

AI has revolutionized the analysis of preoperative imaging studies in plastic surgery. Deep learning algorithms can automatically segment anatomical structures in CT or MRI scans, identify vascular networks for flap planning, and detect abnormalities that might affect surgical outcomes [35].

For complex craniofacial reconstruction, AI-powered software can generate precise three-dimensional models from imaging data, allowing surgeons to plan osteotomies, plate placement, and soft tissue adjustments with unprecedented accuracy [36]. These models can be used to create custom implants or cutting guides, reducing operative time and improving outcomes. The integration of AI with additive manufacturing (3D printing) has enabled the rapid production of patient-specific models, templates, and implants for reconstructive procedures [37].

Vascular mapping for perforator flaps has been significantly enhanced by AI algorithms that can automatically identify suitable perforator vessels from CTA or MRA studies [38]. These systems reduce the time required for preoperative planning while improving the reliability of vessel identification, potentially decreasing operative time and flap complications [39].

### 2.4. Decision Support Systems and Surgical Planning

Comprehensive AI-powered decision support systems integrate multiple aspects of preoperative planning to guide surgeon decision-making. These platforms combine patient-specific risk assessment, outcome prediction, and anatomical analysis to recommend optimal surgical approaches [40].

In breast reconstruction, machine learning algorithms can analyze patient factors, surgical history, and anatomical characteristics to recommend the most appropriate reconstruction technique, whether implant-based or autologous [41]. The system might suggest specific flap types based on the patient’s body habitus and available donor sites while accounting for factors such as radiation history and tissue quality.

For facial reconstruction, AI systems can analyze defect characteristics and available donor tissues to suggest reconstructive options that optimize both functional and aesthetic outcomes [42]. These recommendations consider factors such as tissue match, scar visibility, and functional requirements specific to the defect location.

Beyond technique selection, AI platforms can assist in detailed operative planning, including incision placement, tissue dissection sequences, and anticipated challenges [43]. Some systems incorporate virtual reality or augmented reality interfaces that allow surgeons to rehearse procedures in a three-dimensional environment, potentially reducing operative time and improving outcomes, particularly for less-experienced surgeons [44].

### 2.5. Integration into Clinical Workflow

The successful implementation of AI in preoperative planning depends on seamless integration into existing clinical workflows [45]. User-friendly interfaces, interoperability with electronic health records, and minimal additional time requirements are essential for adoption in busy plastic surgery practices.

Cloud-based platforms allow surgeons to access AI tools from any location, facilitating remote consultations and collaborative planning sessions [46]. Mobile applications enable patient engagement with outcome simulations, enhancing the shared decision-making process [47]. Integration with scheduling systems allows for automated resource allocation based on procedure complexity as determined by AI analysis [48].

As these systems mature, they are increasingly incorporating automated documentation features, generating preoperative plans that can be directly integrated into the medical record. This not only improves efficiency but also creates standardized documentation that may have medicolegal benefits and facilitate retrospective outcome analysis.

The ongoing development of these preoperative planning tools represents a major advance in the practice of plastic surgery, potentially improving surgical precision, patient satisfaction, and clinical outcomes across the spectrum of reconstructive and aesthetic procedures.

### 2.6. Comparative Analysis of AI Preoperative Planning Approaches

The literature on AI applications in preoperative planning for plastic surgery reveals significant methodological and performance variations across different approaches. Table 1 provides a comparative analysis of key studies in this domain, highlighting differences in algorithm types, validation methods, and clinical outcomes.

Artificial intelligence (AI) is increasingly being integrated into preoperative planning within plastic surgery, offering enhanced precision, prediction capabilities, and individualized treatment strategies. This comparative analysis synthesizes findings from ten recent studies exploring various AI-driven approaches across multiple subfields of plastic surgery, including breast reconstruction, rhinoplasty, craniofacial surgery, and microsurgical procedures.

Several studies utilized machine learning and predictive modeling to aid surgical planning. Shoham et al. (2025) [49] implemented a retrospective machine learning model to forecast postoperative complications in breast reduction surgery, achieving high accuracy with a dataset of 322 patients. Similarly, Lim et al. (2024) applied AI to improve radiological interpretation in DIEP flap planning using CT angiography, demonstrating improved learning outcomes for surgical trainees [38].

Other investigations focused on 3D simulation and facial modeling. Eldaly et al. (2022) conducted a systematic review of AI-driven simulation techniques in rhinoplasty, highlighting the growing role of virtual visualization tools in aesthetic planning [31]. Arjmand et al. (2023) presented a predictive AI model capable of estimating soft tissue facial appearance based on craniofacial bone structure, representing a significant advancement in personalized surgical modeling [50].

Review-based studies by Cevik et al. (2023) [51], Nogueira et al. (2025) [52], and Adegboye et al. (2024) [13] emphasized the conceptual and systemic applications of AI in plastic surgery. These works identified emerging trends in the use of AI for flap selection, symmetry prediction, and aesthetic outcome assessments. However, they also pointed out limitations such as the lack of standardized performance metrics, small sample sizes, and limited external validation across studies.

While the diversity in algorithm types—ranging from deep learning and mathematical modeling to computer vision—is noteworthy, a common theme across all studies is the potential of AI to augment clinical decision-making. Nonetheless, limitations such as reliance on retrospective data, the need for prospective trials, and variability in validation methods must be addressed before AI can be widely adopted in clinical practice.

Overall, this comparative analysis underscores AI’s transformative potential in surgical planning while also highlighting the need for standardized frameworks and larger clinical datasets to support its effective integration into routine surgical workflows [53,54,55].

**Table 1:** Comparison of AI preoperative planning studies in plastic surgery.

Study	Algorithm Type	Clinical Application	Sample Size	Validation Method	Primary Outcome	Performance Metrics	Limitations
O’Neill et al. (2020) [22]	Decision-tree with ROSE oversampling	Breast reconstruction flap failure prediction	1012 patients	Testing cohort	Flap failure prediction	AUC: 0.95 (training), 0.67 (testing)	Reduced sensitivity in testing cohort
Cevik et al. (2023) [51]	Machine learning	Breast reconstruction (flap prediction)	N/A (review/conceptual)	Narrative review	AI’s role in improving pre-op planning	Not applicable	Conceptual scope; lacks empirical validation
Lim et al. (2024) [38]	Computer vision + AI	DIEP flap planning (CT angiography)	6 LLMs answering questions	Plastic surgeon panel	Improved learning and vessel interpretation	Diagnostic accuracy	Limited to one training environment
Shoham et al. (2025) [49]	Machine learning (retrospective model)	Breast reduction complication prediction	322 patients	Retrospective analysis	Prediction of post-op complications	AUC, sensitivity, specificity	Retrospective, needs prospective validation
Nogueira et al. (2025) [52]	Deep learning, ML	Aesthetic surgery—general	N/A (systematic review)	Systematic literature review	Qualitative summary of AI applications	Not applicable	Lacks performance aggregation
Eldaly et al. (2022) [31]	Simulation + AI	Rhinoplasty	24 studies reviewed	Systematic review	Visualization and prediction accuracy	Simulation accuracy	Heterogeneity among reviewed studies
Arjmand et al. (2023) [50]	Predictive modeling (AI)	Craniomaxillofacial surgery	5 computed tomography imaging datasets	Model vs. actual face outcome	Face shape prediction from bone	Shape congruence score	Small dataset, complex anatomy modeling
Raj et al. (2024) [56]	Mathematical AI tool	Pre-op rhinoplasty	250 images	Comparison with expert ratings	Objective deformity quantification	Error reduction rate	Nasal deformity focus only
Kapila et al. (2024) [57]	AI (systematic classification)	Microsurgery planning	N/A (systematic review)	Structured classification	6 AI microsurgery domains identified	Not applicable	Narrative; lacks quantitative synthesis
Adegboye et al. (2024) [13]	AI narrative review	Facial plastic and reconstructive surgery	N/A (narrative review)	Descriptive analysis	Categorization of AI applications	Not applicable	No experimental validation
Lanzano (2024) [58]	AI-based modeling	Breast aesthetic planning	N/A (ahead of print, preliminary data)	Conceptual framework	Aesthetic ideal prediction	Not reported	Early stage; no clinical testing yet

## 3. AI-Assisted Robotics in Plastic Surgery

The integration of artificial intelligence with robotic systems represents a significant technological frontier in plastic surgery [14,59]. While robotic platforms have been utilized in various surgical disciplines for decades, their application in plastic and reconstructive procedures has been more limited, primarily due to the complex, three-dimensional nature of soft tissue manipulation and the importance of tactile feedback [60]. However, recent advances in AI algorithms, computer vision, and robotic design are overcoming these barriers, creating new possibilities for AI-assisted robotic systems in plastic surgery.

### 3.1. Current Robotic Systems and Applications

Robotic platforms are increasingly being adapted for plastic surgical procedures, with AI enhancement providing improved precision and autonomy [61]. Initially developed for other surgical specialties, systems like the da Vinci Surgical System [62] have been repurposed for specific plastic surgery applications, including head and neck reconstruction, lymphedema surgery, and selected microsurgical procedures.

The primary advantages of these robotic systems include enhanced visualization through high-definition 3D imaging, tremor filtration, and motion scaling that allows for precise micro-movements [63]. When augmented with AI capabilities, these systems can provide real-time tissue identification, anatomical landmark recognition, and even personalized surgical guidance based on preoperative planning data.

Several specialized robotic systems have emerged specifically for plastic surgery applications. For hair restoration, AI-powered robotic systems can identify optimal hair follicles for harvesting, plan ideal recipient site distribution, and perform follicular unit extraction with remarkable precision and consistency [64]. Similarly, specialized robotic platforms for facial plastic surgery leverage AI to provide guidance for the optimal placement of incisions based on individual facial anatomy and anticipated tissue responses [65].

### 3.2. Machine Learning for Surgical Precision and Automation

The integration of machine learning algorithms with robotic systems has significantly enhanced their capabilities in plastic surgery. Computer vision algorithms can analyze the surgical field in real time, identifying critical structures such as blood vessels and nerves, potentially reducing iatrogenic injury risk [66]. This “surgical GPS” functionality is particularly valuable in anatomically complex regions or in cases of distorted anatomy following trauma or previous surgery.

Reinforcement learning algorithms have enabled robotic systems to continuously improve their performance through iterative feedback, allowing them to adapt to different tissue types and surgical scenarios [60]. These systems can analyze their own performance data across hundreds or thousands of procedures, identifying patterns and optimal approaches that might not be apparent even to experienced surgeons.

In terms of automation, AI has enabled varying levels of autonomous function in surgical robotics. Current clinical systems predominantly operate in a “master–slave” configuration, where the surgeon maintains direct control while the AI provides guidance and assistance. However, research platforms are exploring semi-autonomous functions for specific tasks such as suturing [67,68], where the AI can execute standardized motions while adapting to individual tissue characteristics and tension requirements.

#### Underlying Mechanisms and Technical Foundations

The successful integration of AI with robotic systems in plastic surgery relies on several interconnected technical mechanisms. At the foundation of these systems are computer vision algorithms, typically convolutional neural networks (CNNs), which process visual input from surgical cameras to identify anatomical structures, tissue boundaries, and instrument positions with sub-millimeter precision [69]. These algorithms require extensive training on diverse surgical datasets to account for the significant variability in human anatomy and pathology.

The robotic control systems employ sophisticated reinforcement learning models that translate surgeon input into precise mechanical actions while filtering out physiological tremor and scaling movements [70]. These systems operate on complex biomechanical models that predict tissue behavior during manipulation, considering properties such as elasticity, tensile strength, and fluid dynamics that vary significantly across different tissue types and patient demographics.

The haptic feedback mechanisms utilize a combination of force sensors and machine learning algorithms that translate mechanical resistance into meaningful tactile information [71]. This represents a particularly challenging technical hurdle as the algorithms must bridge the gap between the quantitative sensor data and the qualitative tactile experience that surgeons rely upon for tissue assessment.

These technical foundations explain both the current capabilities and limitations of AI-robotic systems in plastic surgery, where the complexity of soft tissue manipulation presents unique challenges compared to other surgical disciplines that operate on more structurally consistent tissue types.

### 3.3. Microsurgical Applications and Enhancement

Microsurgery represents a particularly promising domain for AI-robotic integration in plastic surgery due to the extreme precision requirements and the limitations of human physiological capabilities [72]. Super-microsurgical procedures involving vessels less than 0.8 mm in diameter can benefit significantly from robotic assistance enhanced by AI algorithms [73].

AI-powered robotic systems for microsurgery incorporate advanced image processing to identify optimal vessel geometry for anastomosis, predict blood flow patterns, and recommend ideal suture placement [74]. Machine learning algorithms can analyze microscopic images in real time to assess vessel quality, predict thrombosis risk, and guide intraoperative decision-making regarding anastomotic technique [75].

Some experimental systems incorporate automated vessel anastomosis capabilities, where the AI guides robotic instruments to perform technically perfect anastomoses with mathematically optimal suture spacing and tension [76]. This technology demonstrates the potential for AI to enhance microsurgical outcomes by exceeding human technical limitations.

Lymphatic surgery, including lymphovenous anastomosis and vascularized lymph node transfer for lymphedema treatment, has also benefited from AI-enhanced robotic platforms [77]. These systems can identify lymphatic vessels as small as 0.1 mm in diameter using near-infrared imaging and specialized machine learning algorithms, dramatically improving the feasibility and efficiency of these technically demanding procedures.

### 3.4. Haptic Feedback and Sensory Augmentation

One of the significant limitations of robotic surgery has been the lack of tactile feedback, which is crucial in plastic surgery for assessing tissue quality, tension, and planes of dissection. Advanced AI algorithms are now addressing this challenge through sensory augmentation and virtual haptic feedback systems [78].

Force sensors integrated into robotic instruments generate data that AI algorithms translate into haptic feedback delivered to the surgeon through specialized interfaces. More sophisticated systems use machine learning to correlate visual tissue deformation with expected tactile sensations, creating “virtual haptics” even in the absence of direct force sensing [79].

Cognitive augmentation represents another frontier, where AI systems analyze multiple data streams (visual, haptic, and physiological) to provide the surgeon with integrated information beyond what human senses could perceive [80]. For example, these systems might overlay perfusion data onto the visual field, alerting the surgeon to compromised tissue viability before it would be visually apparent.

### 3.5. Training and Simulation Systems

AI has transformed robotic surgical training through sophisticated simulation platforms that provide realistic, personalized learning experiences [81]. These systems use machine learning to create physics-based tissue models that accurately replicate the behavior of different tissues during manipulation, cutting, and suturing.

Virtual reality training systems enhanced by AI can adapt to the learner’s skill level, identifying specific weaknesses and generating customized scenarios to address them [82]. Performance metrics derived from expert surgeons serve as benchmarks, with AI algorithms providing objective assessment and feedback on technical skills such as motion efficiency, instrument control, and tissue handling.

Some advanced platforms incorporate “virtual preceptors”—AI systems trained on thousands of procedures performed by expert surgeons that can provide real-time guidance and feedback during training [83]. These systems identify deviations from optimal technique and suggest corrections, potentially accelerating the learning curve for complex robotic procedures.

### 3.6. Challenges and Integration with Human Expertise

The learning curve for surgeons transitioning to robotic platforms is substantial, typically requiring 20–30 cases to achieve proficiency, with studies showing temporarily increased complication rates during this transition period [74]. Additionally, the tactile feedback limitations of current systems remain a significant concern for the nuanced tissue handling required in plastic surgery, with surgeons reporting difficulties in assessing tissue tension and plane development compared to direct manual manipulation.

Regulatory frameworks for increasingly autonomous surgical systems continue to evolve slowly, creating uncertainty around approval pathways and liability considerations [75,76]. Questions regarding responsibility for adverse outcomes remain unresolved when AI systems contribute to surgical decision-making, with current legal frameworks poorly equipped to address these novel scenarios.

Evidence supporting improved outcomes with robotic approaches in plastic surgery remains limited compared to other surgical disciplines. The few comparative studies available show modest benefits in selected applications like microvascular anastomosis precision but often with increased operative time and cost without clear demonstration of superior functional or aesthetic outcomes that would justify widespread adoption [77].

The most effective approach appears to be collaborative, with AI systems enhancing human capabilities rather than replacing them. The concept of “collaborative intelligence” emphasizes the complementary strengths of human surgeons (creativity, adaptability, and ethical judgment) and AI systems (precision, consistency, and data processing). This balanced perspective recognizes both the transformative potential of AI-robotics and the irreplaceable value of human surgical expertise, particularly in a field where artistic judgment and adaptability to unique patient circumstances remain essential.

Despite its promise, AI-assisted robotic surgery in plastic surgery faces substantial challenges that temper optimistic projections with practical reality [84]. Cost remains a significant barrier, with acquisition expenses for robotic systems often exceeding USD 2 million, plus annual maintenance costs of USD 100,000–150,000, placing these technologies beyond the reach of many practices outside large academic or corporate settings [85]. Technical limitations present equally significant obstacles. Current systems remain bulky, with complex setup procedures that can add 30–45 min to operative time, creating workflow disruptions and limiting adoption for shorter procedures where the time–benefit ratio becomes unfavorable. Additionally, regulatory frameworks for increasingly autonomous surgical systems are still evolving, creating uncertainty around approval pathways and liability considerations [86,87].

The optimal integration of AI-robotic systems with human surgical expertise remains a central question. The most effective approach appears to be collaborative, with AI systems enhancing human capabilities rather than replacing them. The concept of “collaborative intelligence” emphasizes the complementary strengths of human surgeons (creativity, adaptability, and ethical judgment) and AI systems (precision, consistency, and data processing).

As these technologies continue to evolve, plastic surgeons must actively participate in their development and implementation to ensure they address genuine clinical needs rather than technological possibilities alone. The future of AI-assisted robotics in plastic surgery will likely involve increasingly sophisticated synergy between human judgment and artificial intelligence, potentially transforming technical capabilities and expanding the boundaries of what is surgically possible.

## 4. AI in Postoperative Care and Patient Monitoring

The application of artificial intelligence in the postoperative phase of plastic surgery represents a rapidly evolving domain with significant potential to improve patient outcomes, reduce complications, and enhance the efficiency of follow-up care. As healthcare systems increasingly prioritize outpatient and ambulatory procedures, the need for sophisticated remote monitoring and early intervention tools becomes more critical. AI technologies are uniquely positioned to address these needs through continuous data analysis, pattern recognition, and predictive modeling [88].

### 4.1. Remote Monitoring Systems and Early Complication Detection

AI-powered remote monitoring represents one of the most promising applications in postoperative care for plastic surgery patients [89]. These systems leverage a combination of wearable sensors, smartphone applications, and machine learning algorithms to track various physiological parameters and detect early signs of complications.

Smart wound dressings embedded with biosensors can monitor temperature, pH, pressure, and moisture levels, transmitting data to AI systems that analyze patterns indicative of infection, hematoma, or compromised perfusion [90]. Computer vision applications allow patients to capture wound images with their smartphones, with AI algorithms assessing healing progression, detecting concerning changes in appearance, and distinguishing between normal postoperative changes and early complications [91].

For free flap monitoring, implantable or wearable doppler systems connected to AI platforms can continuously track blood flow, with algorithms detecting subtle changes that may precede vascular compromise [92]. These systems can alert the surgical team to potential flap failure hours before clinical signs would become apparent, potentially improving salvage rates through earlier intervention.

The integration of these monitoring tools with electronic health records creates comprehensive patient profiles that allow AI algorithms to contextualize individual data points within the patient’s overall clinical picture. This holistic analysis enables more accurate risk stratification and personalized monitoring protocols based on procedure type, patient comorbidities, and individual recovery patterns.

### 4.2. Predictive Analytics for Complication Risk

Beyond real-time monitoring, AI systems excel at predicting complication risks during the recovery period by analyzing diverse data inputs [93]. These predictive models integrate preoperative risk factors, intraoperative events, and postoperative monitoring data to identify patients at elevated risk for specific complications [94].

Machine learning algorithms can predict the likelihood of surgical site infections by analyzing factors such as operative time, tissue perfusion measurements, wound closure technique, and postoperative vital signs [95]. Similar models exist for predicting seroma formation, dehiscence, and flap compromise, allowing for targeted preventive interventions in high-risk patients.

More sophisticated systems incorporate psychosocial factors and behavioral data to predict adherence to postoperative instructions, which significantly impacts recovery outcomes. By identifying patients at risk for non-adherence, these algorithms enable proactive interventions such as additional education, more frequent follow-up, or enhanced support systems [96].

These predictive platforms continue to refine their accuracy through continuous learning, analyzing outcomes data across thousands of patients to identify novel risk factors and improve prediction specificity. Some systems now incorporate genetic and molecular markers, further personalizing risk assessment and enabling precision approaches to postoperative care.

### 4.3. Pain Management and Prescription Optimization

AI applications have shown particular promise in optimizing postoperative pain management, a critical aspect of recovery in plastic surgery. Machine learning algorithms can analyze individual patient characteristics, genetic factors, and procedure-specific variables to predict pain response patterns and medication requirements [97].

These systems can recommend personalized analgesic regimens based on predicted needs, potentially reducing both inadequate pain control and excessive opioid prescription. By continuously analyzing patient-reported pain scores, medication consumption patterns, and physiological parameters, AI platforms can suggest appropriate adjustments to pain management strategies in real time [98].

In the context of the opioid epidemic, AI tools that predict individual opioid requirements have particular value [99]. These algorithms identify patients who may achieve adequate pain control with minimal or no opioid medications, as well as those who might benefit from enhanced multimodal analgesia or closer monitoring due to elevated risk factors for persistent use [100].

Natural language processing applications can analyze patient communications (through secure messaging systems or conversational AI interfaces) to identify language patterns associated with inadequate pain control, enabling earlier intervention. Similarly, these systems can recognize linguistic indicators of potential medication misuse, supporting responsible prescribing practices [101].

### 4.4. Patient Engagement and Adherence Tools

Patient engagement and adherence to postoperative instructions significantly impact outcomes in plastic surgery. AI technologies have created new opportunities to enhance engagement through personalized communication, education, and motivation systems [102].

Conversational AI applications (chatbots and virtual assistants) provide patients with 24/7 access to information and support, answering questions about normal recovery, activity restrictions, and wound care. These systems use natural language processing to interpret patient queries and provide contextually appropriate responses, escalating concerns to human providers when necessary [103].

Personalized education platforms use machine learning to adapt content based on individual learning styles, health literacy levels, and specific procedure details. These systems deliver information in digestible formats at optimal times throughout the recovery process, rather than overwhelming patients with all instructions at discharge [104].

Gamification elements powered by behavioral prediction algorithms can improve adherence to activity restrictions, compression garment use, and other postoperative recommendations. By providing personalized goals, progress tracking, and positive reinforcement, these applications leverage behavioral psychology principles to optimize recovery behaviors [105].

### 4.5. Outcome Assessment and Long-Term Monitoring

The assessment of surgical outcomes represents another area where AI technologies offer significant advantages in plastic surgery. Computer vision and machine learning algorithms can analyze standardized postoperative images to quantify aesthetic results with unprecedented objectivity and consistency [106].

Three-dimensional imaging systems coupled with AI analysis can assess volumetric changes, symmetry, contour, and other aesthetic parameters, creating objective metrics for outcomes that have traditionally relied on subjective evaluation [107]. These technologies enable more rigorous comparative studies of different surgical techniques and more accurate assessment of long-term results.

For functional outcomes, AI systems can analyze movement patterns, the range of motion, and other performance metrics captured through smartphone sensors or specialized devices. This quantitative approach to functional assessment allows for early detection of suboptimal results and timely intervention before problematic scar formation or other issues become difficult to address [108].

Long-term monitoring applications leverage periodic patient-submitted images and questionnaire data to track outcomes over years rather than months [109]. Machine learning algorithms can detect patterns of change that might indicate issues such as implant malposition, fat graft resorption, or other late complications, prompting appropriate follow-up.

### 4.6. Integration with Quality Improvement Systems

Perhaps the most transformative potential of AI in postoperative care lies in its ability to continuously analyze outcomes data across large patient populations, identifying opportunities for quality improvement at both individual surgeon and institutional levels [110].

AI systems can analyze postoperative complication rates, patient satisfaction scores, and other outcome metrics, correlating these with specific surgical techniques, perioperative protocols, or surgeon characteristics. This analysis can identify evidence-based best practices and areas for potential improvement that might not be apparent through traditional quality assessment methods [11].

Automated analysis of patient-reported outcome measures (PROMs) using natural language processing can extract nuanced insights from qualitative feedback, identifying specific aspects of care that drive satisfaction or dissatisfaction [111]. These insights can inform targeted improvements in both technical and interpersonal aspects of care delivery.

For individual surgeons, AI-powered surgical analytics platforms can provide personalized performance dashboards comparing their outcomes to anonymized peer benchmarks, with specific recommendations for technique modifications or practice changes to improve results [112]. This data-driven approach to professional development complements traditional continuing education and may accelerate the adoption of best practices.

As these systems mature, they create feedback loops that continuously refine plastic surgical care, with insights from postoperative data informing modifications to preoperative planning and intraoperative technique. This cycle of continuous improvement, powered by AI analysis of comprehensive outcomes data, has the potential to significantly advance the field beyond what could be achieved through traditional clinical research methods alone [113].

## 5. Challenges and Ethical Considerations

The integration of artificial intelligence into plastic surgery presents numerous challenges and raises important ethical questions that must be addressed to ensure responsible implementation. While the potential benefits are substantial, thoughtful consideration of these concerns is essential to safeguard patient welfare, maintain professional standards, and preserve the human elements of surgical care that are central to the specialty [114].

### 5.1. Data Privacy and Security

Plastic surgery involves particularly sensitive patient data, including detailed images of patients’ bodies, personal health information, and, in many cases, psychological assessments. The collection, storage, and processing of these data for AI applications raises significant privacy concerns that extend beyond standard medical confidentiality requirements [115].

The use of preoperative and postoperative images for training AI algorithms requires robust anonymization protocols, but complete de-identification can be challenging, particularly for facial images or distinctive anatomical features. Even with advanced anonymization techniques, there remains a risk of re-identification through correlation with other data sources or through the unique patterns that AI systems might detect [116].

Cloud-based AI platforms, which offer computational advantages for complex algorithms, introduce additional security considerations regarding data transmission and storage. International variations in data protection regulations create further complications for multinational research collaborations and global AI implementation [117].

Patient consent for AI applications presents unique challenges as the nature of machine learning means that future uses of data may not be fully predictable at the time of collection [118]. Developing ethical frameworks for broad consent that respects patient autonomy, while enabling beneficial technological advancement remains an ongoing challenge.

### 5.2. Algorithmic Bias and Fairness

AI systems are only as unbiased as the data used to train them, and significant concerns exist regarding representational fairness in plastic surgery algorithms. Historical disparities in access to plastic surgical care have resulted in databases that may underrepresent certain demographic groups, potentially leading to algorithms that perform differently across populations [119].

This issue is particularly relevant in aesthetic applications, where beauty standards vary across cultures and where training data may reflect narrow or culturally specific ideals. AI systems trained predominantly on certain racial or ethnic groups may produce biased recommendations or predictions when applied to patients from different backgrounds [120].

Even when demographic diversity is present in training data, underlying inequities in healthcare access and quality may be encoded in the algorithms, potentially perpetuating or amplifying these disparities. For example, if complication rates in training data are higher for certain populations due to social determinants of health rather than intrinsic risk factors, AI systems might incorrectly assess risk based on demographic characteristics.

Addressing these concerns requires diverse and representative training datasets, careful attention to potential bias in algorithm development, and ongoing monitoring of AI performance across different patient populations. Transparent reporting of algorithm limitations and performance characteristics is essential for ethical implementation [121].

### 5.3. Implementation Challenges in Clinical Practice

Beyond theoretical considerations, the practical integration of AI into daily plastic surgery practice presents substantial challenges that have received insufficient attention in the literature. Resistance from healthcare providers represents a significant barrier, stemming from various sources including skepticism about AI performance, concerns about disruption to established workflows, and fears regarding professional autonomy or eventual replacement [122]. Studies in other surgical specialties have documented adoption rates as low as 20–30% for certain AI tools even when provided at no cost, highlighting the importance of addressing these attitudinal barriers [123].

The training requirements for effective AI utilization are considerable and often underestimated. Surgeons must develop not only basic technical competency with these systems but also the critical evaluation skills necessary to appropriately interpret AI outputs and recommendations. This requires substantial time investment from busy clinicians who may not see immediate returns on this educational effort. Current plastic surgery training programs provide limited formal education in AI concepts, creating a knowledge gap that impedes informed adoption [124].

Workflow integration presents another significant challenge. Many AI systems operate as standalone tools rather than components of integrated clinical information systems, requiring additional steps that disrupt clinical efficiency. Over-reliance on AI systems poses additional risks, potentially leading to skill degradation in tasks delegated to AI or creating unwarranted trust in algorithmic recommendations. This “automation bias” has been documented in other medical contexts, where clinicians sometimes defer to technological outputs even when they contradict clinical judgment or contain obvious errors [125]. In a field requiring refined technical skills and aesthetic judgment, such as plastic surgery, this phenomenon raises particular concerns.

Technical infrastructure limitations present practical obstacles in many practice settings. AI systems often require robust computing resources, high-bandwidth network connectivity, and specialized technical support that may not be available outside academic medical centers or large healthcare systems. Physical space constraints in operating rooms and clinics may also complicate the integration of additional technological systems into already crowded environments.

Addressing these implementation challenges requires a sociotechnical approach that addresses not only the technological aspects of AI but also the human, organizational, and systemic factors that influence adoption. User-centered design methodologies, robust change management strategies, and attention to workflow optimization are essential for the successful integration of AI into routine plastic surgery practice.

### 5.4. Professional Liability and Decision-Making Authority

The integration of AI into clinical decision-making creates complex questions regarding professional liability and the appropriate balance between algorithmic recommendations and surgeon judgment [126]. As AI systems become more sophisticated in predicting surgical outcomes or recommending treatment approaches, determining responsibility for adverse outcomes becomes increasingly complicated [127].

If a surgeon follows an AI recommendation that leads to a suboptimal result, questions arise regarding liability distribution between the surgeon, the AI developer, and potentially other stakeholders. Conversely, if a surgeon disregards an AI recommendation that later proves accurate, they may face questions about failure to utilize available technology appropriately [128].

Current legal frameworks are not fully adapted to address these scenarios, and professional standards for appropriate reliance on AI guidance are still evolving [129]. The concept of the surgeon as the ultimate decision-maker may require refinement as AI capabilities advance, potentially shifting toward a model of collaborative intelligence where responsibility is shared between human and artificial systems.

For these reasons, transparent documentation of AI-assisted decision-making processes becomes crucial for medicolegal purposes. Systems that provide explainable recommendations, rather than inscrutable “black box” outputs, will be essential for responsible integration into clinical practice.

### 5.5. Impact on Surgeon–Patient Relationship

Plastic surgery has traditionally involved a deeply personal relationship between surgeon and patient, with significant emphasis on trust, communication, and shared decision-making [130]. The introduction of AI technologies has the potential to either enhance or disrupt this relationship, depending on implementation approaches [131].

On one hand, AI tools can provide surgeons with more accurate information and predictions to share with patients, potentially improving the informed consent process [132]. These technologies can also free surgeons from routine computational tasks, allowing more time for meaningful patient interaction.

However, excessive reliance on technology may create distance between surgeon and patient, particularly if AI systems replace rather than augment human communication [133]. Patients may feel that their care has become less personalized if they perceive treatment decisions to be algorithmically driven rather than tailored to their individual needs and preferences by a human surgeon.

The aesthetic aspect of plastic surgery involves subjective judgments and artistic sensibility that may not be fully captured by AI systems. Maintaining the balance between technological advancement and the human elements of surgical care presents an ongoing challenge for the specialty.

### 5.6. Regulatory Frameworks and Validation Standards

The rapidly evolving nature of AI technologies has outpaced regulatory frameworks, creating uncertainty regarding approval pathways, validation requirements, and implementation standards [134]. Current medical device regulations were not designed with machine learning algorithms in mind, particularly those with “adaptive” capabilities that continue to evolve after deployment.

Establishing appropriate validation methodologies for AI applications in plastic surgery remains challenging. Traditional clinical trial designs may not be well suited to evaluate continuously learning systems, and appropriate comparators for novel AI applications may not exist. The balance between innovation and evidence-based implementation requires careful consideration.

International harmonization of regulatory approaches represents another challenge as divergent requirements across jurisdictions may impede the global adoption of beneficial technologies [135]. Collaborative efforts between regulatory bodies, professional societies, and industry stakeholders are essential to develop appropriate oversight mechanisms that ensure patient safety without unnecessarily impeding innovation [136].

### 5.7. Educational and Implementation Barriers

The integration of AI into plastic surgery practice requires significant educational efforts to prepare both current practitioners and trainees for this technological evolution [137]. Most practicing plastic surgeons have limited formal training in data science, creating potential barriers to appropriate evaluation and implementation of AI tools [138].

Curriculum development for plastic surgery training programs must evolve to incorporate fundamental data science concepts, AI evaluation skills, and the critical appraisal of algorithmic outputs [139]. This educational challenge extends to allied health professionals, patients, and healthcare administrators who interact with these technologies.

Implementation barriers include not only knowledge gaps but also practical considerations such as cost, workflow integration, and technological infrastructure requirements. Disparities in access to advanced technologies may exacerbate existing inequities in healthcare delivery if implementation occurs predominantly in well-resourced settings [140].

### 5.8. Commercialization and Conflicts of Interest

The commercial potential of AI applications in plastic surgery creates possibilities for conflicts of interest that may influence development priorities, validation approaches, and implementation decisions [141]. The market-driven nature of many AI initiatives may favor applications with commercial appeal rather than those addressing the most significant clinical needs.

Partnerships between academic institutions and industry actors, while potentially beneficial for innovation, require careful management to maintain scientific integrity and prioritize patient welfare. Transparency regarding financial relationships and development methodologies is essential for maintaining trust in these technologies [142].

The proprietary nature of many commercial AI systems creates challenges for independent validation and comparison across platforms. Closed algorithms that cannot be scrutinized by independent researchers raise concerns regarding scientific validation and ongoing quality assurance.

### 5.9. Toward Responsible Innovation

Despite these challenges, the potential benefits of AI in plastic surgery warrant continued development with appropriate safeguards. A framework for responsible innovation must balance technological advancement with ethical considerations, incorporating principles such as transparency, fairness, accountability, and respect for patient autonomy [143].

Multidisciplinary collaboration between plastic surgeons, data scientists, ethicists, patient advocates, and regulatory experts can help navigate these complex issues. Professional societies have an important role in developing guidelines, standards, and educational resources to support appropriate AI implementation.

Ultimately, the goal should be to harness the potential of AI to enhance surgical care while preserving the human judgment, artistic sensibility, and ethical foundations that define the specialty of plastic surgery. By thoughtfully addressing these challenges, the field can navigate the AI revolution in a manner that prioritizes patient welfare and advances the specialty’s core missions of restoration, reconstruction, and aesthetic enhancement [144].

### 5.10. Limitations of Current Research and Implementation

The integration of AI into plastic surgery faces several important limitations that warrant acknowledgment. First, much of the current research on AI applications in plastic surgery relies on retrospective analyses with relatively small, single-institution datasets that may not generalize across diverse patient populations and practice settings. Many studies report promising results in controlled environments but lack validation in prospective, multi-center trials that would more accurately reflect real-world clinical conditions.

From a methodological perspective, the field faces challenges in standardizing outcome measures and evaluation metrics, making direct comparisons between different AI approaches difficult. The subjective nature of many aesthetic outcomes further complicates objective assessment of AI performance in these applications. Additionally, many studies focus on technical feasibility and algorithm performance rather than demonstrating meaningful improvements in patient outcomes or cost-effectiveness.

Implementation research remains particularly limited, with few studies addressing the practical challenges of integrating AI tools into existing clinical workflows, electronic health record systems, and reimbursement models. The economic implications of AI adoption, including initial investment costs, maintenance requirements, and potential return on investment, have not been thoroughly investigated in the context of plastic surgery practice.

Finally, there is a notable gap in research examining patient perspectives on AI use in plastic surgery, including preferences regarding disclosure, comfort levels with different applications, and cultural variations in acceptance of these technologies. These limitations highlight the need for more robust, patient-centered research approaches as the field continues to evolve.

## 6. Critical Analysis of the Evidence Base

The evaluation of AI applications in plastic surgery requires careful consideration of the quality and strength of supporting evidence. This section critically examines the current evidence base, highlighting both promising findings and significant limitations.

### 6.1. Strength of Current Evidence

The evidence supporting AI applications in plastic surgery varies considerably across different domains. The strongest evidence exists for imaging analysis applications, particularly in areas such as skin lesion classification, where deep learning algorithms have demonstrated performance comparable to board-certified dermatologists in controlled studies with sensitivity and specificity exceeding 90% [145]. These applications benefit from the availability of large, well-annotated image datasets and clear ground-truth diagnoses.

Preoperative risk assessment represents another area with relatively robust evidence. Several machine learning models for predicting complications following breast reconstruction have been externally validated in multi-institutional cohorts, demonstrating consistent AUC values between 0.70 and 0.78 [146]. These models have shown superior performance compared to traditional risk calculators, though implementation studies examining their impact on clinical decision-making and patient outcomes remain limited.

For AI-assisted robotics, evidence is largely limited to technical feasibility studies and case series rather than comparative trials. The few available comparative studies show mixed results, with some demonstrating improved precision in specific tasks like microvascular anastomosis, but others showing no significant differences in overall outcomes compared to conventional approaches [147].

### 6.2. Methodological Limitations

Critical methodological limitations affect much of the current literature on AI in plastic surgery. Sample size and representativeness concerns are prominent, with many studies utilizing convenience samples from single institutions that may not reflect the diversity of patient populations. Studies frequently exclude complex or atypical cases, limiting generalizability to routine practice where such cases are encountered regularly.

Validation approaches often rely on retrospective data splitting rather than prospective or external validation, potentially overestimating real-world performance. When external validation is performed, performance metrics typically decrease by 10–15%, highlighting the challenge of developing generalizable algorithms [148].

Outcome definitions and evaluation metrics vary widely across studies, complicating meaningful comparison and synthesis of evidence. This inconsistency is particularly problematic for aesthetic applications, where standardized, objective outcome measures remain elusive.

Control group selection introduces additional concerns, with many studies comparing AI performance to trainees rather than experienced surgeons or using historical controls without accounting for potential confounding variables. Few studies employ randomized designs that would provide more definitive evidence of effectiveness.

### 6.3. Translation Gap

A significant translation gap exists between algorithm development and clinical implementation. Despite numerous publications demonstrating promising algorithm performance in controlled research environments, few AI applications in plastic surgery have progressed to widespread clinical use. This gap results from multiple factors, including the following:

1. Limited prospective validation in routine clinical environments.
2. Insufficient evidence of cost-effectiveness or outcome improvement.
3. Implementation barriers including workflow integration challenges.
4. Regulatory hurdles and uncertainty.
5. Lack of reimbursement mechanisms for AI-assisted procedures.

The predominance of feasibility and proof-of-concept studies without subsequent implementation research creates a “valley of death” between initial development and clinical adoption. Recent systematic reviews have identified numerous promising algorithms without corresponding studies examining their performance in routine clinical use [149].

This critical analysis of the evidence base highlights the need for more robust, patient-centered research approaches as AI applications in plastic surgery continue to evolve. Moving beyond algorithm development to implementation science, comparative effectiveness research, and health economic evaluation represents an essential next step for the field.

## 7. Future Directions

As artificial intelligence continues to evolve at an accelerating pace, its integration into plastic surgery stands at a critical juncture. The preceding sections have detailed current applications across the surgical timeline, from preoperative planning through postoperative care, as well as the significant challenges these technologies present. This final section explores emerging developments and future trajectories that may shape the next generation of AI applications in plastic surgery, concluding with a perspective on the specialty’s path forward in the age of artificial intelligence.

### 7.1. Emerging Technologies and Methodologies

Several technological frontiers show particular promise for advancing AI capabilities in plastic surgery over the coming decade. Multimodal AI systems that integrate diverse data types—including images, text, physiological measurements, and genetic information—are likely to provide more comprehensive and nuanced insights than current models focused on single data modalities [150]. These systems may better capture the multifaceted nature of surgical decision-making, where visual, tactile, and contextual information all inform clinical judgment.

Federated learning approaches offer a potential solution to data privacy concerns while enabling the large-scale collaboration necessary for robust algorithm development [151]. By allowing AI models to be trained across multiple institutions without sharing raw patient data, these methods may accelerate innovation while protecting sensitive information. Edge computing architectures that process data locally on devices rather than in centralized clouds further enhance privacy protections while enabling real-time analysis for time-sensitive applications [152].

Explainable AI (XAI) [153] represents another crucial frontier, developing algorithms that provide transparent rationales for their recommendations rather than functioning as inscrutable “black boxes”. As regulatory requirements increasingly emphasize algorithmic transparency, these approaches will be essential for clinical adoption and may enhance surgeon trust in AI recommendations.

The integration of AI with extended reality (XR) technologies—including augmented, virtual, and mixed reality—creates new possibilities for surgical planning, intraoperative guidance, and training [154]. AI algorithms can enhance XR experiences by providing context-aware information, predicting optimal visualization perspectives, and personalizing educational content to individual learning needs.

### 7.2. Generative AI Applications

Generative AI represents a particularly promising frontier for plastic surgery applications. Unlike previous AI systems focused primarily on classification or prediction, generative models can create novel content based on learned patterns. This capability has significant implications for surgical planning and simulation.

Advanced generative adversarial networks (GANs) and diffusion models could enhance surgical outcome simulation beyond current capabilities, generating photorealistic visualizations of potential results that account for individual anatomical variation, tissue properties, and healing characteristics [155]. These technologies could substantially improve the informed consent process by helping patients visualize and understand potential outcomes with unprecedented realism.

For reconstructive applications, generative AI could assist in designing custom implants and tissue constructs optimized for individual patient anatomy and functional requirements. By generating multiple design iterations and simulating their biomechanical performance, these systems could identify optimal solutions that might not be apparent through traditional design approaches [156].

Text-to-image and text-to-3D generative models could transform preoperative communication between patients and surgeons. Patients could describe desired aesthetic outcomes in natural language, with AI systems generating visual representations that serve as a starting point for discussion, potentially reducing misalignment between patient expectations and achievable results [157].

In education and training, generative AI could create infinite variations of surgical scenarios for simulation, allowing trainees to encounter rare complications or anatomical variations that would be difficult to experience during normal training. These synthetic training environments could adapt to individual learning needs, providing personalized educational experiences [158].

### 7.3. Real-Time Data Integration and Adaptive Systems

Future AI systems will likely move beyond static models to dynamic, adaptive platforms that continuously incorporate new data to refine their performance. Real-time integration of intraoperative data—including physiological monitoring, tissue perfusion assessment, and surgical field visualization—with preoperative planning could enable adaptive surgical navigation that updates recommendations based on evolving conditions [159].

These adaptive systems would represent a significant advance over current approaches that rely on preoperative data alone, allowing for adjustment to unexpected findings or complications. For example, in free flap procedures, real-time perfusion data could be integrated with anatomical models to suggest alternative anastomosis sites if primary vessels prove unsuitable during surgery.

Continuous learning systems that evolve based on outcomes data represent another frontier. Rather than requiring periodic retraining with curated datasets, these systems would incrementally refine their algorithms based on each new case, gradually improving performance while maintaining appropriate validation safeguards [160]. This approach could accelerate the translation of research advances into clinical practice and enable more personalized recommendations based on institution-specific patient populations.

### 7.4. Integration with Other Digital Health Technologies

The true transformative potential of AI in plastic surgery may lie not in isolated applications but in its convergence with other emerging healthcare technologies. The integration of AI with the Internet of Medical Things (IoMT) creates opportunities for continuous monitoring across the entire patient journey, from preoperative optimization through long-term follow-up [161]. Smart surgical instruments that collect and analyze data during procedures may enable real-time adjustment of surgical technique based on tissue response and predicted outcomes [162].

Digital twins—virtual models of individual patients that simulate physiological responses—represent another frontier where AI may significantly impact surgical planning [163]. These models could allow surgeons to test multiple surgical approaches virtually before selecting the optimal intervention for a specific patient, potentially improving outcomes while reducing reoperation rates.

Blockchain technologies may address data provenance and consent management challenges in AI development, creating immutable records of data usage permissions and algorithm training history. This transparency could enhance patient trust while facilitating responsible data sharing for research and development [164].

The integration of AI with regenerative medicine approaches, including 3D bioprinting and tissue engineering, may enable more personalized reconstructive solutions [165]. AI algorithms could optimize scaffold design based on individual anatomy, predict cell behavior in engineered tissues, and guide the development of patient-specific implants with improved functional and aesthetic outcomes.

### 7.5. Evolution of Clinical Practice and Business Models

As AI technologies mature, they will likely catalyze significant changes in plastic surgery practice models and care delivery approaches. Distributed surgical planning, where specialists collaborate remotely on complex cases supported by AI analysis, may improve access to expertise regardless of geographic location. Similarly, AI-enhanced telemedicine platforms may extend the reach of plastic surgical care to underserved populations through remote assessment and follow-up [166].

Business models in plastic surgery will likely adapt to incorporate AI capabilities, potentially shifting from purely fee-for-service approaches toward value-based models where outcomes prediction and risk stratification play central roles [167]. Practice efficiency enhancements through AI automation may allow surgeons to focus on the most complex aspects of care while algorithmic systems manage routine tasks [168].

The relationship between plastic surgeons and technology developers will continue to evolve, with increasing emphasis on collaborative design processes that incorporate clinical expertise from the earliest stages of development. Surgeon entrepreneurs may play important roles in this ecosystem, bridging clinical needs with technological capabilities through start-up ventures and industry partnerships.

### 7.6. Research Priorities and Knowledge Gaps

Despite significant progress, substantial knowledge gaps remain in the application of AI to plastic surgery [169]. Critical research priorities include the development of standardized validation methodologies for surgical AI applications, the creation of more diverse and representative training datasets, and the rigorous evaluation of AI impact on clinical outcomes beyond technical performance metrics.

Implementation science research examining the factors that influence successful AI adoption in clinical settings represents another priority. Studies exploring the optimal integration of AI capabilities into surgical workflow, as well as the educational approaches that best prepare surgeons to work effectively with these technologies, will be essential for realizing their potential benefits.

Ethical frameworks for AI use in specific plastic surgery contexts require further development, particularly regarding applications in aesthetic surgery where definitions of successful outcomes may be more subjective [170]. Research examining patient perspectives on AI use, including preferences regarding disclosure and comfort with algorithmic involvement in different aspects of care, will inform responsible implementation approaches.

Long-term studies assessing the impact of AI-assisted decision-making on surgeon skill development and clinical judgment represent another crucial area for investigation. Understanding how reliance on AI tools affects surgical training and capability maintenance will be essential for designing educational programs that maintain core surgical competencies while embracing technological advancement.

### 7.7. Personalized AI-Driven Interventions

The future of AI in plastic surgery will likely emphasize increasingly personalized approaches that account for individual patient characteristics, preferences, and outcomes. Current risk prediction models typically classify patients into broad risk categories based on population-level data, but future systems may generate truly individualized recommendations based on comprehensive patient profiles [171].

These personalized models would integrate conventional clinical data with novel inputs including genetic information, tissue biomechanical properties, microbiome profiles, and even psychosocial factors to generate highly specific predictions and recommendations. This approach aligns with the broader trend toward precision medicine, where treatments are tailored to individual characteristics rather than population averages. Beyond technical personalization, AI systems may increasingly incorporate patient preferences and values into their recommendations. Preference-sensitive decision support tools could help surgeons and patients navigate complex trade-offs between different surgical approaches, accounting for individual priorities regarding factors such as recovery time, scarring, functional outcomes, and aesthetic results [172].

This evolution toward personalized, preference-sensitive AI support represents a potential solution to concerns about algorithmic standardization diminishing the individualized nature of plastic surgical care. Rather than imposing uniform approaches, these systems would enhance the surgeon’s ability to provide truly patient-centered care.

### 7.8. Preparing the Plastic Surgery Workforce

Successfully navigating the AI revolution will require deliberate efforts to prepare the plastic surgery workforce for this technological transformation. Curriculum development for residency programs should incorporate foundational data science concepts, critical appraisal of AI research, and hands-on experience with relevant applications [173].

Continuing education programs for practicing surgeons must address both technical understanding and implementation considerations, delivered in formats that accommodate diverse learning preferences and technology comfort levels [174]. Recognizing that not all surgeons will develop specialized AI expertise, educational approaches should focus on creating “informed consumers” who can appropriately evaluate and implement these tools in their practice.

Interdisciplinary training experiences that bring together plastic surgery trainees with data scientists, engineers, and ethicists may foster the collaborative skills necessary for the next generation of innovation. Similarly, involving patients in educational initiatives can ensure that human-centered perspectives remain central to technology development and implementation.

Professional societies have a critical role in developing practice guidelines, ethical frameworks, and quality standards for AI applications in plastic surgery [175]. These organizations can also facilitate knowledge sharing and collaborative research initiatives that advance the evidence base for clinical implementation.

## 8. Conclusions: Shaping the Future of Plastic Surgery in the Age of AI

Artificial intelligence represents perhaps the most significant technological force shaping the future of plastic surgery in the coming decades. The applications described throughout this review demonstrate the potential for AI to enhance surgical precision, improve outcome prediction, and expand the boundaries of what is surgically possible. At the same time, the challenges discussed highlight the need for thoughtful implementation that preserves the human elements of surgical care and addresses important ethical considerations.

The path forward requires balanced perspective—neither uncritical enthusiasm that overlooks legitimate concerns nor excessive caution that foregoes meaningful benefits for patients. Plastic surgeons must actively engage with AI development rather than merely responding to technologies created without their input, ensuring that these tools address genuine clinical needs and align with the specialty’s core values.

The fundamental goals of plastic surgery—the restoration of form and function, the alleviation of suffering, and the enhancement of the quality of life—remain constant even as technological capabilities evolve. Artificial intelligence should be viewed not as a replacement for surgical judgment and technical skill but as a powerful tool that can augment human capabilities and help fulfill these enduring missions.

By embracing responsible innovation, addressing implementation challenges, and maintaining focus on patient-centered care, plastic surgery can navigate the AI revolution in a manner that honors its rich traditions while embracing new possibilities. The integration of artificial and human intelligence in surgical practice offers the promise of care that is simultaneously more precise and more personal and more evidence-based and more compassionate—truly representing the best of both technological advancement and the humanistic tradition of medicine.

## Data Availability

Data are available at reasonable request.

## Abbreviations

The following abbreviations are used in this manuscript:

AI	Artificial intelligence
ML	Machine learning
DL	Deep learning
NLP	Natural language processing
CT	Computed tomography
MRI	Magnetic resonance imaging
CTA	Computed tomography angiography
MRA	Magnetic resonance angiography
3D	Three-dimensional
XAI	Explainable artificial intelligence
XR	Extended reality
IOMT	Internet of Medical Things
PROMs	Patient-reported outcome measures

## References

[1] Zyoud S.H. (2025). A Bibliometric Analysis of the Integration of mHealth, Telemedicine, and OpenAI in Aesthetic Plastic Surgery: Advancements, Trends, and Future Directions. Aesthetic Plast. Surg..

[2] Landau M., Kassir M., Goldust M. (2025). Transforming Aesthetic Medicine with Generative Artificial Intelligence. J. Cosmet. Dermatol..

[3] Hao Y., Shan M., Liu H., Xia Y., Kuang X., Song K., Wang Y. (2025). Comparison of Predictive Models for Keloid Recurrence Based on Machine Learning. J. Cosmet. Dermatol..

[4] Qiu X., Zhong C., Chen Q., Zhao Y., Yang T., Zhou J., Liao S., Chen J. (2025). Zygomatic Osteotomy surgery design software based on skull CT scans—Self-supervised algo reduces workload. J. Cranio-Maxillofac. Surg..

[5] Mahedia M., Rohrich R.N., Sadiq K.O., Bailey L., Harrison L.M., Hallac R.R. (2025). Exploring the Utility of ChatGPT in Cleft Lip Repair Education. J. Clin. Med..

[6] Jarvis T., Thornburg D., Rebecca A.M., Teven C.M. (2020). Artificial Intelligence in Plastic Surgery: Current Applications, Future Directions, and Ethical Implications. Plast. Reconstr. Surg. Glob. Open.

[7] Zhang S., Zhang Y., Ouyang X., Li H., Dai R. (2025). Random forest algorithm for predicting postoperative hypotension in oral cancer resection and free flap reconstruction surgery. Sci. Rep..

[8] Lopes J., Guimarães T., Duarte J., Santos M. (2025). Enhancing Surgery Scheduling in Health Care Settings with Metaheuristic Optimization Models: Algorithm Validation Study. JMIR Med. Inform..

[9] Knoedler L., Hoch C.C., Knoedler S., Klimitz F.J., Schaschinger T., Niederegger T., Heiland M., Koerdt S., Pooth R., Kauke-Navarro M. (2025). Objectifying aesthetic outcomes following face transplantation—The AI research metrics model (CAARISMA ® ARMM). J. Stomatol. Oral. Maxillofac. Surg..

[10] Perlmutter J.W., Milkovich J., Fremont S., Datta S., Mosa A. (2025). Beyond the Surface: Assessing GPT-4’s Accuracy in Detecting Melanoma and Suspicious Skin Lesions from Dermoscopic Images. Plast Surg..

[11] Guni A., Varma P., Zhang J., Fehervari M., Ashrafian H. (2024). Artificial Intelligence in Surgery: The Future Is Now. Eur. Surg. Res..

[12] Buccheri E.M., Villanucci A. (2025). Response to: Letter on Artificial Intelligence: Enhancing Scientific Presentations in Aesthetic Surgery. Aesthetic Plast. Surg..

[13] Adegboye F.O., Peterson A.A., Sharma R.K., Stephan S.J., Patel P.N., Yang S.F. (2024). Applications of Artificial Intelligence in Facial Plastic and Reconstructive Surgery: A Narrative Review. Facial Plast. Surg. Aesthetic Med..

[14] Barone M., De Bernardis R., Persichetti P. (2024). Artificial Intelligence in Plastic Surgery: Analysis of Applications, Perspectives, and Psychological Impact. Aesthetic Plast. Surg..

[15] Seth I., Lim B., Joseph K., Gracias D., Xie Y., Ross R.J., Rozen W.M. (2024). Use of artificial intelligence in breast surgery: A narrative review. Gland. Surg..

[16] Gorgy A., Xu H.H., Hawary H.E., Nepon H., Lee J., Vorstenbosch J. (2024). Integrating AI into Breast Reconstruction Surgery: Exploring Opportunities, Applications, and Challenges. Plast. Surg..

[17] Sarikonda A., Abishek R., Isch E.L., Momin A.A., Self M., Sambangi A., Carreras A., Jallo J., Harrop J., Sivaganesan A. (2024). Assessing the Clinical Appropriateness and Practical Utility of ChatGPT as an Educational Resource for Patients Considering Minimally Invasive Spine Surgery. Cureus.

[18] Kolivand M., Al-jumeily D., Miraz M.H., Southall G., Ali M., Ware A. (2024). Pre-planning for Plastic Surgery Using Machine Learning: A Proof of Concept. Emerging Technologies in Computing.

[19] Vles M.D., Terng N.C.O., Zijlstra K., Mureau M.A.M., Corten E.M.L. (2020). Virtual and augmented reality for preoperative planning in plastic surgical procedures: A systematic review. J. Plast. Reconstr. Aesthetic Surg..

[20] Abi-Rafeh J., Bassiri-Tehrani B., Kazan R., Furnas H., Hammond D., Adams W.P., Nahai F. (2024). Preoperative Patient Guidance and Education in Aesthetic Breast Plastic Surgery: A Novel Proposed Application of Artificial Intelligence Large Language Models. Aesthetic Surg. J. Open Forum.

[21] Ghanem M., Ghaith A.K., Bydon M., Bydon M. (2024). Chapter 6—Artificial intelligence and personalized medicine: Transforming patient care. The New Era of Precision Medicine.

[22] O’Neill A.C., Yang D., Roy M., Sebastiampillai S., Hofer S.O.P., Xu W. (2020). Development and Evaluation of a Machine Learning Prediction Model for Flap Failure in Microvascular Breast Reconstruction. Ann. Surg. Oncol..

[23] Naoum G.E., Ho A.Y., Shui A., Salama L., Goldberg S., Arafat W., Winograd J., Colwell A., Smith B.L., Taghian A.G. (2022). Risk of Developing Breast Reconstruction Complications: A Machine-Learning Nomogram for Individualized Risk Estimation with and without Postmastectomy Radiation Therapy. Plast. Reconstr. Surg..

[24] Braun S.E., Sinik L.M., Meyer A.M., Larson K.E., Butterworth J.A. (2023). Predicting Complications in Breast Reconstruction: Development and Prospective Validation of a Machine Learning Model. Ann. Plast. Surg..

[25] Tan I.J., Katamanin O.M., Greene R.K., Jafferany M. (2024). Artificial intelligence in psychodermatology: A brief report of applications and impact in clinical practice. Skin. Res. Technol..

[26] Sutton R.T., Pincock D., Baumgart D.C., Sadowski D.C., Fedorak R.N., Kroeker K.I. (2020). An overview of clinical decision support systems: Benefits, risks, and strategies for success. NPJ Digit. Med..

[27] Varghese C., Harrison E.M., O’Grady G., Topol E.J. (2024). Artificial intelligence in surgery. Nat. Med..

[28] Park J.J., Tiefenbach J., Demetriades A.K. (2022). The role of artificial intelligence in surgical simulation. Front. Med. Technol..

[29] Lin Z., Lei C., Yang L. (2023). Modern Image-Guided Surgery: A Narrative Review of Medical Image Processing and Visualization. Sensors.

[30] Brenac C., Fazilat A.Z., Fallah M., Kawamoto-Duran D., Sunwoo P.S., Longaker M.T., Wan D.C., Guo J.L. (2024). AI in plastic surgery: Customizing care for each patient. AIS.

[31] Eldaly A.S., Avila F.R., Torres-Guzman R.A., Maita K., Garcia J.P., Palmieri Serrano L., Forte A.J. (2022). Simulation and Artificial Intelligence in Rhinoplasty: A Systematic Review. Aesthetic Plast. Surg..

[32] Zhang B.H., Chen K., Lu S.M., Nakfoor B., Cheng R., Gibstein A., Tanna N., Thorne C.H., Bradley J.P. (2021). Turning Back the Clock: Artificial Intelligence Recognition of Age Reduction after Face-Lift Surgery Correlates with Patient Satisfaction. Plast. Reconstr. Surg..

[33] Seth I., Bulloch G., Joseph K., Hunter-Smith D.J., Rozen W.M. (2023). Use of Artificial Intelligence in the Advancement of Breast Surgery and Implications for Breast Reconstruction: A Narrative Review. J. Clin. Med..

[34] Kim J., Lee S.M., Kim D.E., Kim S., Chung M.J., Kim Z., Kim T., Lee K.-T. (2024). Development of an Automated Free Flap Monitoring System Based on Artificial Intelligence. JAMA Netw. Open.

[35] Wendler T., van Leeuwen F.W.B., Navab N., van Oosterom M.N. (2021). How molecular imaging will enable robotic precision surgery. Eur. J. Nucl. Med. Mol. Imaging.

[36] Fadero P.E., Shah M. (2014). Three dimensional (3D) modelling and surgical planning in trauma and orthopaedics. Surgeon.

[37] Shaikh S., Shaikh S. (2024). 3D Printing in Surgical Planning: 3D Reconstruction and Surgical Guides. 3D Printing in Prosthetics and Orthotics: Innovations and Opportunities.

[38] Lim B., Cevik J., Seth I., Sofiadellis F., Ross R.J., Rozen W.M., Cuomo R. (2024). Evaluating Artificial Intelligence’s Role in Teaching the Reporting and Interpretation of Computed Tomographic Angiography for Preoperative Planning of the Deep Inferior Epigastric Artery Perforator Flap. JPRAS Open.

[39] Moosa S., Dydynsky R. (2022). The Role of Artificial Intelligence in Predicting Flap Outcomes in Plastic Surgery: Protocol of a Systematic Review. Undergrad. Res. Nat. Clin. Sci. Technol. J..

[40] Patel A., Dada A., Saggi S., Yamada H., Ambati V.S., Goldstein E., Hsiao E.C., Mummaneni P.V. (2024). Personalized Approaches to Spine Surgery. Int. J. Spine Surg..

[41] Ospina-Gómez J.E., Molano-Diaz J.M., Rojas-Gómez M.C., Latorre-Arévalo M.G., Sanchez-Vargas M. (2025). Advances and applications of artificial intelligence in breast reconstruction surgery: A systematic review. Eur. J. Plast. Surg..

[42] Tokgöz E., Carro M.A., Tokgöz E., Carro M.A. (2023). Applications of Artificial Intelligence, Machine Learning, and Deep Learning on Facial Plastic Surgeries. Cosmetic and Reconstructive Facial Plastic Surgery: A Review of Medical and Biomedical Engineering and Science Concepts.

[43] Dundar T.T., Yurtsever I., Pehlivanoglu M.K., Yildiz U., Eker A., Demir M.A., Mutluer A.S., Tektaş R., Kazan M.S., Kitis S. (2022). Machine Learning-Based Surgical Planning for Neurosurgery: Artificial Intelligent Approaches to the Cranium. Front. Surg..

[44] Kim Y., Kim H., Kim Y.O. (2017). Virtual Reality and Augmented Reality in Plastic Surgery: A Review. Arch. Plast. Surg..

[45] Thaker N.G., Loaiza-Bonilla A., Choudhri A., Doria C. (2004). Bringing AI to the Clinic for Pancreatic Cancer Care: The Imperative for Seamless Integration into Clinical Workflows. AI Precis. Oncol..

[46] Monteiro A.C.B., França R.P., Arthur R., Iano Y., Siarry P., Jabbar M.A., Aluvalu R., Abraham A., Madureira A. (2021). An Overview of Medical Internet of Things, Artificial Intelligence, and Cloud Computing Employed in Health Care from a Modern Panorama. The Fusion of Internet of Things, Artificial Intelligence, and Cloud Computing in Health Care.

[47] Shih H.-J., Xue H., Min H., Wojtusiak J., Chang J. (2023). Informing Patient-Provider Engagement for Shared Decision Making Through Mobile Health Applications. PPA.

[48] Knight D.R.T., Aakre C.A., Anstine C.V., Munipalli B., Biazar P., Mitri G., Valery J.R., Brigham T., Niazi S.K., Perlman A.I. (2023). Artificial intelligence for patient scheduling in the real-world health care setting: A metanarrative review. Health Policy Technol..

[49] Shoham G., Zuckerman T., Fliss E., Govrin O., Zaretski A., Singolda R., Kedar D.J., Leshem D., Madah E., Arad E. (2025). Utilizing Artificial Intelligence for Predicting Postoperative Complications in Breast Reduction Surgery: A Comprehensive Retrospective Analysis of Predictive Features and Outcomes. Aesthetic Surg. J..

[50] Arjmand H., Clement A., Hardisty M., Fialkov J.A., Whyne C.M. (2023). Artificial Intelligence–Based Modeling Can Predict Face Shape Based on Underlying Craniomaxillofacial Bone. J. Craniofacial Surg..

[51] Cevik J., Seth I., Rozen W.M. (2023). Transforming breast reconstruction: The pioneering role of artificial intelligence in preoperative planning. Gland. Surg..

[52] Nogueira R., Eguchi M., Kasmirski J., de Lima B.V., Dimatos D.C., Lima D.L., Glatter R., Tran D.L., Piccinini P.S. (2025). Machine Learning, Deep Learning, Artificial Intelligence and Aesthetic Plastic Surgery: A Qualitative Systematic Review. Aesthetic Plast. Surg..

[53] Rodler S., Ganjavi C., De Backer P., Magoulianitis V., Ramacciotti L.S., De Castro Abreu A.L., Gill I.S., Cacciamani G.E. (2024). Generative artificial intelligence in surgery. Surgery.

[54] RKato M., Parizotto J.D.O.L., Oliveira P.H.J.D., Gonçalves J.R. (2023). Artificial Intelligence in Orthognathic Surgery—A Narrative Review of Surgical Digital Tools and 3D Orthognathic Surgical Planning. J. Calif. Dent. Assoc..

[55] Shujaat S., Riaz M., Jacobs R. (2023). Synergy between artificial intelligence and precision medicine for computer-assisted oral and maxillofacial surgical planning. Clin. Oral Investig..

[56] Raj U., Garg A., Vathulya M., Kandwal A. (2024). Quantifying nasal deformities using a novel mathematical method to complement preoperative assessment in rhinoplasty patients. J. Plast. Reconstr. Aesthetic Surg..

[57] Kapila A.K., Georgiou L., Hamdi M. (2024). Decoding the Impact of AI on Microsurgery: Systematic Review and Classification of Six Subdomains for Future Development. Plast. Reconstr. Surg. Glob. Open.

[58] Lanzano G. (2024). The Role of AI in Crafting Aesthetically Ideal Breasts: Enhancing Preoperative Planning in Plastic Surgery. Aesthetic Plast. Surg..

[59] Murphy D., Saleh D. (2020). Artificial Intelligence in plastic surgery: What is it? Where are we now? What is on the horizon?. Annals.

[60] Struk S., Qassemyar Q., Leymarie N., Honart J.-F., Alkhashnam H., De Fremicourt K., Conversano A., Schaff J.-B., Rimareix F., Kolb F. (2018). The ongoing emergence of robotics in plastic and reconstructive surgery. Ann. Chir. Plast. Esthétique.

[61] Duong T.V., Vy V.P.T., Hung T.N.K. (2024). Artificial Intelligence in Plastic Surgery: Advancements, Applications, and Future. Cosmetics.

[62] Azizian M., Liu M., Khalaji I., Sorger J., Oh D., Daimios S., Abedin-Nasab M.H. (2020). 3—The da Vinci Surgical System. Handbook of Robotic and Image-Guided Surgery.

[63] Wee I.J.Y., Kuo L.-J., Ngu J.C.-Y. (2020). A systematic review of the true benefit of robotic surgery: Ergonomics. Int. J. Med. Robot. Comput. Assist. Surg..

[64] Gupta A.K., Ivanova I.A., Renaud H.J. (2021). How good is artificial intelligence (AI) at solving hairy problems? A review of AI applications in hair restoration and hair disorders. Dermatol. Ther..

[65] Tokgöz E., Carro M.A., Tokgöz E., Carro M.A. (2023). Robotics Applications in Facial Plastic Surgeries. Cosmetic and Reconstructive Facial Plastic Surgery: A Review of Medical and Biomedical Engineering and Science Concepts.

[66] Ward T.M., Mascagni P., Ban Y., Rosman G., Padoy N., Meireles O., Hashimoto D.A. (2021). Computer vision in surgery. Surgery.

[67] Datta S., Li Y., Ruppert M.M., Ren Y., Shickel B., Ozrazgat-Baslanti T., Rashidi P., Bihorac A. (2021). Reinforcement learning in surgery. Surgery.

[68] Ostrander B.T., Massillon D., Meller L., Chiu Z.-Y., Yip M., Orosco R.K. (2024). The current state of autonomous suturing: A systematic review. Surg. Endosc..

[69] Pfeiffer M., Riediger C., Weitz J., Speidel S. (2019). Learning soft tissue behavior of organs for surgical navigation with convolutional neural networks. Int. J. CARS.

[70] Kumar A., Kumar S., Kaushik A., Kumar A., Saini J.S. (2022). Real time estimation and suppression of hand tremor for surgical robotic applications. Microsyst. Technol..

[71] Bergholz M., Ferle M., Weber B.M. (2023). The benefits of haptic feedback in robot assisted surgery and their moderators: A meta-analysis. Sci. Rep..

[72] Thamm O.C., Eschborn J., Schäfer R.C., Schmidt J. (2024). Advances in Modern Microsurgery. J. Clin. Med..

[73] Hong J.P., Song S., Suh H.S.P. (2018). Supermicrosurgery: Principles and applications. J. Surg. Oncol..

[74] Savage S.A., Seth I., Angus Z.G., Rozen W.M. (2025). Advancements in microsurgery: A comprehensive systematic review of artificial intelligence applications. J. Plast. Reconstr. Aesthetic Surg..

[75] Tang M., Sugiyama T., Takahari R., Sugimori H., Yoshimura T., Ogasawara K., Kudo K., Fujimura M. (2024). Assessment of changes in vessel area during needle manipulation in microvascular anastomosis using a deep learning-based semantic segmentation algorithm: A pilot study. Neurosurg. Rev..

[76] Marcus H.J., Ramirez P.T., Khan D.Z., Layard Horsfall H., Hanrahan J.G., Williams S.C., Beard D.J., Bhat R., Catchpole K., Cook A. (2024). The IDEAL framework for surgical robotics: Development, comparative evaluation and long-term monitoring. Nat. Med..

[77] Eldaly A.S., Avila F.R., Torres-Guzman R.A., Maita K., Garcia J.P., Serrano L.P., Forte A.J. (2022). Artificial intelligence and lymphedema: State of the art. J. Clin. Transl. Res..

[78] Knudsen J.E., Ghaffar U., Ma R., Hung A.J. (2024). Clinical applications of artificial intelligence in robotic surgery. J. Robot. Surg..

[79] Minopoulos G.M., Memos V.A., Stergiou K.D., Stergiou C.L., Psannis K.E. (2023). A Medical Image Visualization Technique Assisted with AI-Based Haptic Feedback for Robotic Surgery and Healthcare. Appl. Sci..

[80] Dias R.D., Yule S.J., Zenati M.A., Atallah S. (2021). Augmented Cognition in the Operating Room. Digital Surgery.

[81] Iftikhar M., Saqib M., Zareen M., Mumtaz H. (2024). Artificial intelligence: Revolutionizing robotic surgery: Review. Ann. Med. Surg..

[82] Kazemzadeh K., Akhlaghdoust M., Zali A. (2023). Advances in artificial intelligence, robotics, augmented and virtual reality in neurosurgery. Front. Surg..

[83] Sinha A., West A., Vasdev N., Sooriakumaran P., Rane A., Dasgupta P., McKirdy M. (2023). Current practises and the future of robotic surgical training. Surgeon.

[84] Wah J.N.K. (2025). Revolutionizing surgery: AI and robotics for precision, risk reduction, and innovation. J. Robot. Surg..

[85] Byrd J.K., Paquin R. (2020). Cost Considerations for Robotic Surgery. Otolaryngol. Clin. N. Am..

[86] O’Sullivan S., Nevejans N., Allen C., Blyth A., Leonard S., Pagallo U., Holzinger K., Holzinger A., Sajid M.I., Ashrafian H. (2019). Legal, regulatory, and ethical frameworks for development of standards in artificial intelligence (AI) and autonomous robotic surgery. Int. J. Med. Robot. Comput. Assist. Surg..

[87] Lee A., Baker T.S., Bederson J.B., Rapoport B.I. (2024). Levels of autonomy in FDA-cleared surgical robots: A systematic review. NPJ Digit. Med..

[88] Jiang F., Jiang Y., Zhi H., Dong Y., Li H., Ma S., Wang Y., Dong Q., Shen H., Wang Y. (2017). Artificial intelligence in healthcare: Past, present and future. Stroke Vasc. Neurol..

[89] Del Vecchio D., Stein M.J., Dayan E., Marte J., Theodorou S. (2023). Nanotechnology and Artificial Intelligence: An Emerging Paradigm for Postoperative Patient Care. Aesthetic Surg. J..

[90] Prakashan D., Kaushik A., Gandhi S. (2024). Smart sensors and wound dressings: Artificial intelligence-supported chronic skin monitoring—A review. Chem. Eng. J..

[91] Kitaguchi D., Takeshita N., Hasegawa H., Ito M. (2022). Artificial intelligence-based computer vision in surgery: Recent advances and future perspectives. Ann. Gastroenterol. Surg..

[92] Bassani S., Eccher A., Molteni G. (2023). Harnessing the Power of Artificial Intelligence: Revolutionizing Free Flaps Monitoring in Head and Neck Tumor Treatment. CRO.

[93] Stam W.T., Goedknegt L.K., Ingwersen E.W., Schoonmade L.J., Bruns E.R.J., Daams F. (2022). The prediction of surgical complications using artificial intelligence in patients undergoing major abdominal surgery: A systematic review. Surgery.

[94] Bonde A., Varadarajan K.M., Bonde N., Troelsen A., Muratoglu O.K., Malchau H., Yang A.D., Alam H., Sillesen M. (2021). Assessing the utility of deep neural networks in predicting postoperative surgical complications: A retrospective study. Lancet Digit. Health.

[95] Wu G., Khair S., Yang F., Cheligeer C., Southern D., Zhang Z., Feng Y., Xu Y., Quan H., Williamson T. (2022). Performance of machine learning algorithms for surgical site infection case detection and prediction: A systematic review and meta-analysis. Ann. Med. Surg..

[96] Xue B., Li D., Lu C., King C.R., Wildes T., Avidan M.S., Kannampallil T., Abraham J. (2021). Use of Machine Learning to Develop and Evaluate Models Using Preoperative and Intraoperative Data to Identify Risks of Postoperative Complications. JAMA Netw. Open.

[97] Zhang M., Zhu L., Lin S.-Y., Herr K., Chi C.-L., Demir I., Dunn Lopez K., Chi N.-C. (2023). Using artificial intelligence to improve pain assessment and pain management: A scoping review. J. Am. Med. Inform. Assoc..

[98] Fontaine D., Vielzeuf V., Genestier P., Limeux P., Santucci-Sivilotto S., Mory E., Darmon N., Lanteri-Minet M., Mokhtar M., Laine M. (2022). Artificial intelligence to evaluate postoperative pain based on facial expression recognition. Eur. J. Pain.

[99] Wang J.E., Beaulieu-Jones B., Brat G.A., Marwaha J.S. (2024). The role of artificial intelligence in helping providers manage pain and opioid use after surgery. Glob. Surg. Educ..

[100] Gabriel R.A., Park B.H., Hsu C.-N., Macias A.A. (2025). A Review of Leveraging Artificial Intelligence to Predict Persistent Postoperative Opioid Use and Opioid Use Disorder and its Ethical Considerations. Curr. Pain. Headache Rep..

[101] Levy R.A., Kay A.H., Hills N., Chen L., Chapman J.S. (2024). Exploring the relationship between language, postoperative pain, and opioid use. AJOG Glob. Rep..

[102] Campbell K., Louie P., Levine B., Gililland J. (2020). Using Patient Engagement Platforms in the Postoperative Management of Patients. Curr. Rev. Musculoskelet. Med..

[103] Dwyer T., Hoit G., Burns D., Higgins J., Chang J., Whelan D., Kiroplis I., Chahal J. (2023). Use of an Artificial Intelligence Conversational Agent (Chatbot) for Hip Arthroscopy Patients Following Surgery. Arthrosc. Sports Med. Rehabil..

[104] Shahid M., Mehmood R. (2024). AI in Rehabilitation and Physical Therapy: Personalized Recovery Plans for Patients. Int. J. Artif. Intell. Cybersecur..

[105] Kontadakis G., Chasiouras D., Proimaki D., Halkiadakis M., Fyntikaki M., Mania K. (2020). Gamified platform for rehabilitation after total knee replacement surgery employing low cost and portable inertial measurement sensor node. Multimed. Tools Appl..

[106] Mascagni P., Alapatt D., Sestini L., Altieri M.S., Madani A., Watanabe Y., Alseidi A., Redan J.A., Alfieri S., Costamagna G. (2022). Computer vision in surgery: From potential to clinical value. NPJ Digit. Med..

[107] Tzou C.-H.J., Artner N.M., Pona I., Hold A., Placheta E., Kropatsch W.G., Frey M. (2014). Comparison of three-dimensional surface-imaging systems. J. Plast. Reconstr. Aesthetic Surg..

[108] Al-hababi M.A.M., Khan M.B., Al-Turjman F., Zhao N., Yang X. (2020). Non-Contact Sensing Testbed for Post-Surgery Monitoring by Exploiting Artificial-Intelligence. Appl. Sci..

[109] Shaik T., Tao X., Higgins N., Li L., Gururajan R., Zhou X., Acharya U.R. (2023). Remote patient monitoring using artificial intelligence: Current state, applications, and challenges. WIREs Data Min. Knowl. Discov..

[110] Salati M., Migliorelli L., Moccia S., Andolfi M., Roncon A., Guiducci G.M., Xiumè F., Tiberi M., Frontoni E., Refai M. (2021). A Machine Learning Approach for Postoperative Outcome Prediction: Surgical Data Science Application in a Thoracic Surgery Setting. World J. Surg..

[111] Lu Z., Sim J., Wang J.X., Forrest C.B., Krull K.R., Srivastava D., Hudson M.M., Robison L.L., Baker J.N., Huang I.-C. (2021). Natural Language Processing and Machine Learning Methods to Characterize Unstructured Patient-Reported Outcomes: Validation Study. J. Med. Internet Res..

[112] Colborn K., Brat G., Callcut R. (2023). Predictive Analytics and Artificial Intelligence in Surgery—Opportunities and Risks. JAMA Surg..

[113] Hashimoto D.A., Rosman G., Rus D., Meireles O.R. (2018). Artificial Intelligence in Surgery: Promises and Perils. Ann. Surg..

[114] Cobianchi L., Verde J.M., Loftus T.J., Piccolo D., Dal Mas F., Mascagni P., Garcia Vazquez A., Ansaloni L., Marseglia G.R., Massaro M. (2022). Artificial Intelligence and Surgery: Ethical Dilemmas and Open Issues. J. Am. Coll. Surg..

[115] Kenig N., Monton Echeverria J., Rubi C. (2024). Ethics for AI in Plastic Surgery: Guidelines and Review. Aesthetic Plast. Surg..

[116] Choi E., Leonard K.W., Jassal J.S., Levin A.M., Ramachandra V., Jones L.R. (2023). Artificial Intelligence in Facial Plastic Surgery: A Review of Current Applications, Future Applications, and Ethical Considerations. Facial Plast. Surg..

[117] Salako A., Fabuyi J., Aideyan N.T., Selesi-Aina O., Dapo-Oyewole D.L., Olaniyi O.O. (2024). Advancing Information Governance in AI-Driven Cloud Ecosystem: Strategies for Enhancing Data Security and Meeting Regulatory Compliance. Asian J. Res. Comput. Sci..

[118] Kotsenas A.L., Balthazar P., Andrews D., Geis J.R., Cook T.S. (2021). Rethinking Patient Consent in the Era of Artificial Intelligence and Big Data. J. Am. Coll. Radiol..

[119] Baxter N.B., Howard J.C., Chung K.C. (2021). A Systematic Review of Health Disparities Research in Plastic Surgery. Plast. Reconstr. Surg..

[120] Rahman E., Sadeghi-Esfahlani S., Rao P., Garcia P., Ioannidis S., Nosta J., Rahman Z., Webb W.R. (2025). Skin, scalpel and the silicon chip: A systematic review on the accuracy, bias and data governance of artificial intelligence in dermatology, minimally invasive aesthetics, aesthetic, plastic and reconstructive surgery. Eur. J. Plast. Surg..

[121] Kiseleva A., Kotzinos D., De Hert P. (2022). Transparency of AI in Healthcare as a Multilayered System of Accountabilities: Between Legal Requirements and Technical Limitations. Front. Artif. Intell..

[122] Cabitza F., Rasoini R., Gensini G.F. (2017). Unintended Consequences of Machine Learning in Medicine. JAMA.

[123] Topol E.J. (2019). High-performance medicine: The convergence of human and artificial intelligence. Nat. Med..

[124] Kapila A.K., Hamdi M. (2024). Time to Evolve Plastic Surgery Education? Integrating Robotic Techniques and Artificial Intelligence into Training Curricula. Plast. Reconstr. Surg. Glob. Open.

[125] Goddard K., Roudsari A., Wyatt J.C. (2012). Automation bias: A systematic review of frequency, effect mediators, and mitigators. J. Am. Med. Inform. Assoc..

[126] Terranova C., Cestonaro C., Fava L., Cinquetti A. (2024). AI and professional liability assessment in healthcare. A revolution in legal medicine?. Front. Med..

[127] Griffin F. (2021). Artificial Intelligence and Liability in Health Care. Health Matrix.

[128] Jamjoom A.A.B., Jamjoom A.M.A., Thomas J.P., Palmisciano P., Kerr K., Collins J.W., Vayena E., Stoyanov D., Marcus H.J. (2022). The iRobotSurgeon Collaboration Autonomous surgical robotic systems and the liability dilemma. Front. Surg..

[129] Rincon N., Gerke S., Wagner J.K. (2024). Implications of An Evolving Regulatory Landscape on the Development of AI and ML in Medicine. Biocomputing 2025.

[130] Chen K., Congiusta S., Nash I.S., Coppa G.F., Smith M.L., Kasabian A.K., Thorne C., Tanna N. (2018). Factors Influencing Patient Satisfaction in Plastic Surgery: A Nationwide Analysis. Plast. Reconstr. Surg..

[131] Rodler S., Kopliku R., Ulrich D., Kaltenhauser A., Casuscelli J., Eismann L., Waidelich R., Buchner A., Butz A., Cacciamani G.E. (2024). Patients’ Trust in Artificial Intelligence–based Decision-making for Localized Prostate Cancer: Results from a Prospective Trial. Eur. Urol. Focus.

[132] Farid Y., Chang C., Marcasciano M., Meglio F.D., Rodríguez-Mantilla I., Nanni J., Ortiz S., Chen H.-C. (2024). Consent 2.0: Informed choices in the age of artificial intelligence. Surgery.

[133] Adegbesan A., Akingbola A., Aremu O., Adewole O., Amamdikwa J.C., Shagaya U. (2024). From Scalpels to Algorithms: The Risk of Dependence on Artificial Intelligence in Surgery. J. Med. Surg. Public Health.

[134] Khan F. (2023). Regulating the Revolution: A Legal Roadmap to Optimizing AI in Healthcare. Minn. JL Sci. Tech..

[135] Reddy S. (2025). Global Harmonization of Artificial Intelligence-Enabled Software as a Medical Device Regulation: Addressing Challenges and Unifying Standards. Mayo Clin. Proc. Digit. Health.

[136] Cancela-Outeda C. (2024). The EU’s AI act: A framework for collaborative governance. Internet Things.

[137] Kachalia A., Mello M.M., Nallamothu B.K., Studdert D.M. (2016). Legal and Policy Interventions to Improve Patient Safety. Circulation.

[138] Turner A.E., Abu-Ghname A., Davis M.J., Ali K., Winocour S. (2020). Role of Simulation and Artificial Intelligence in Plastic Surgery Training. Plast. Reconstr. Surg..

[139] Spoer D.L., Kiene J.M., Dekker P.K., Huffman S.S., Kim K.G., Abadeer A.I., Fan K.L. (2022). A Systematic Review of Artificial Intelligence Applications in Plastic Surgery: Looking to the Future. Plast. Reconstr. Surg. Glob. Open.

[140] Celi L.A., Cellini J., Charpignon M.-L., Dee E.C., Dernoncourt F., Eber R., Mitchell W.G., Moukheiber L., Schirmer J., Situ J. (2022). Sources of bias in artificial intelligence that perpetuate healthcare disparities—A global review. PLoS Digit. Health.

[141] Karamchandani M.M., Tian T., Hall R., Nickel I., Aalberg J., Lassaletta A.D., Chatterjee A., Walters D.M. (2024). Discrepancies in Financial Conflicts of Interest in Robotic Cardiothoracic Surgery Studies. Ann. Thorac. Surg..

[142] Polevikov S. (2023). Advancing AI in healthcare: A comprehensive review of best practices. Clin. Chim. Acta.

[143] Reddy S., Rogers W., Makinen V.-P., Coiera E., Brown P., Wenzel M., Weicken E., Ansari S., Mathur P., Casey A. (2021). Evaluation framework to guide implementation of AI systems into healthcare settings. BMJ Health Care Inform..

[144] Mennella C., Maniscalco U., Pietro G.D., Esposito M. (2024). Ethical and regulatory challenges of AI technologies in healthcare: A narrative review. Heliyon.

[145] Haenssle H.A., Fink C., Schneiderbauer R., Toberer F., Buhl T., Blum A., Kalloo A., Hassen A.B.H., Thomas L., Enk A. (2018). Man against machine: Diagnostic performance of a deep learning convolutional neural network for dermoscopic melanoma recognition in comparison to 58 dermatologists. Ann. Oncol..

[146] Hassan A.M., Rajesh A., Asaad M., Nelson J.A., Coert J.H., Mehrara B.J., Butler C.E. (2023). Artificial Intelligence and Machine Learning in Prediction of Surgical Complications: Current State, Applications, and Implications. Am. Surg..

[147] Diana M., Marescaux J. (2015). Robotic surgery. Br. J. Surg..

[148] Steinhubl S.R., Topol E.J. (2015). Moving From Digitalization to Digitization in Cardiovascular Care. J. Am. College Cardiol. JACC.

[149] Liu X., Faes L., Kale A.U., Wagner S.K., Fu D.J., Bruynseels A., Mahendiran T., Moraes G., Shamdas M., Kern C. (2019). A comparison of deep learning performance against health-care professionals in detecting diseases from medical imaging: A systematic review and meta-analysis. Lancet Digit. Health.

[150] Haider S.A., Ho O.A., Borna S., Gomez-Cabello C.A., Pressman S.M., Cole D., Sehgal A., Leibovich B.C., Forte A.J. (2025). Use of Multimodal Artificial Intelligence in Surgical Instrument Recognition. Bioengineering.

[151] Wen J., Zhang Z., Lan Y., Cui Z., Cai J., Zhang W. (2023). A survey on federated learning: Challenges and applications. Int. J. Mach. Learn. Cyber..

[152] Klonoff D.C. (2017). Fog Computing and Edge Computing Architectures for Processing Data From Diabetes Devices Connected to the Medical Internet of Things. J. Diabetes Sci. Technol..

[153] Patidar N., Mishra S., Jain R., Prajapati D., Solanki A., Suthar R., Patel K., Patel H. (2024). Transparency in AI Decision Making: A Survey of Explainable AI Methods and Applications. Adv. Robot. Technol..

[154] Zhang J., Lu V., Khanduja V. (2023). The impact of extended reality on surgery: A scoping review. Int. Orthop. SICOT.

[155] Frid-Adar M., Diamant I., Klang E., Amitai M., Goldberger J., Greenspan H. (2018). GAN-based synthetic medical image augmentation for increased CNN performance in liver lesion classification. Neurocomputing.

[156] Moroni L., Burdick J.A., Highley C., Lee S.J., Morimoto Y., Takeuchi S., Yoo J.J. (2018). Biofabrication strategies for 3D in vitro models and regenerative medicine. Nat. Rev. Mater..

[157] Lee H., Savva M., Chang A.X. (2024). Text-to-3D Shape Generation. Comput. Graph. Forum.

[158] Pakkasjärvi N., Luthra T., Anand S. (2023). Artificial Intelligence in Surgical Learning. Surgeries.

[159] Maier-Hein L., Eisenmann M., Sarikaya D., März K., Collins T., Malpani A., Fallert J., Feussner H., Giannarou S., Mascagni P. (2022). Surgical data science—From concepts toward clinical translation. Med. Image Anal..

[160] Nguyen D.C., Pham Q.-V., Pathirana P.N., Ding M., Seneviratne A., Lin Z., Dobre O., Hwang W.-J. (2022). Federated Learning for Smart Healthcare: A Survey. ACM Comput. Surv..

[161] Mulita F., Verras G.-I., Anagnostopoulos C.-N., Kotis K. (2022). A Smarter Health through the Internet of Surgical Things. Sensors.

[162] Crawford M. (2023). TOWARD SMARTER SURGERIES: Surgical instrumentation is being developed with better materials and “smart” technologies, leading to more efficient procedures. Orthop. Des. Technol..

[163] Ahmed H., Devoto L. (2021). The Potential of a Digital Twin in Surgery. Surg. Innov..

[164] Hasselgren A., Rensaa J.-A.H., Kralevska K., Gligoroski D., Faxvaag A. (2021). Blockchain for Increased Trust in Virtual Health Care: Proof-of-Concept Study. J. Med. Internet Res..

[165] Mackay B.S., Marshall K., Grant-Jacob J.A., Kanczler J., Eason R.W., Oreffo R.O.C., Mills B. (2021). The future of bone regeneration: Integrating AI into tissue engineering. Biomed. Phys. Eng. Express.

[166] Huang E.Y., Knight S., Guetter C.R., Davis C.H., Moller M., Slama E., Crandall M. (2019). Telemedicine and telementoring in the surgical specialties: A narrative review. Am. J. Surg..

[167] Tariq A., Gill A.Y., Hussain H.K. (2023). Evaluating the Potential of Artificial Intelligence in Orthopedic Surgery for Value-based Healthcare. Int. J. Multidiscip. Sci. Arts IJMDSA.

[168] Alowais S.A., Alghamdi S.S., Alsuhebany N., Alqahtani T., Alshaya A.I., Almohareb S.N., Aldairem A., Alrashed M., Bin Saleh K., Badreldin H.A. (2023). Revolutionizing healthcare: The role of artificial intelligence in clinical practice. BMC Med. Educ..

[169] Farid Y., Fernando Botero Gutierrez L., Ortiz S., Gallego S., Zambrano J.C., Morrelli H.U., Patron A. (2024). Artificial Intelligence in Plastic Surgery: Insights from Plastic Surgeons, Education Integration, ChatGPT’s Survey Predictions, and the Path Forward. Plast. Reconstr. Surg. Glob. Open.

[170] Ching S., Thoma A., McCabe R.E., Antony M.M. (2003). Measuring Outcomes in Aesthetic Surgery: A Comprehensive Review of the Literature. Plast. Reconstr. Surg..

[171] Hamamoto R., Suvarna K., Yamada M., Kobayashi K., Shinkai N., Miyake M., Takahashi M., Jinnai S., Shimoyama R., Sakai A. (2020). Application of Artificial Intelligence Technology in Oncology: Towards the Establishment of Precision Medicine. Cancers.

[172] He J., Baxter S.L., Xu J., Xu J., Zhou X., Zhang K. (2019). The practical implementation of artificial intelligence technologies in medicine. Nat. Med..

[173] Wang R., Lucy A., Cochrun S., Abraham P., Hardiman K.M., Corey B., Chen H. (2023). Preserving the Pipeline of Surgeon Scientists: The Role of a Structured Research Curriculum. J. Surg. Res..

[174] Agrawal N., Turner A., Grome L., Abu-Ghname A., Davis M.J., Reece E.M., Buchanan E.P., Winocour S. (2020). Use of Simulation in Plastic Surgery Training. Plast. Reconstr. Surg. Glob. Open.

[175] Webb W.R., Rao P., Garcia P.E., Carruthers J.D.A., Rahman E. (2024). Harmony and hype: Navigating translational science in aesthetic medicine and plastic surgery. Eur. J. Plast. Surg..

